# Freshwater mussel conservation: A global horizon scan of emerging threats and opportunities

**DOI:** 10.1111/gcb.16510

**Published:** 2022-11-29

**Authors:** David C. Aldridge, Isobel S. Ollard, Yulia V. Bespalaya, Ivan N. Bolotov, Karel Douda, Juergen Geist, Wendell R. Haag, Michael W. Klunzinger, Manuel Lopes‐Lima, Musa C. Mlambo, Nicoletta Riccardi, Ronaldo Sousa, David L. Strayer, Santiago H. Torres, Caryn C. Vaughn, Tadeusz Zając, Alexandra Zieritz

**Affiliations:** ^1^ Aquatic Ecology Group, Department of Zoology University of Cambridge Cambridge UK; ^2^ N. Laverov Federal Center for Integrated Arctic Research of the Ural Branch of the Russian Academy of Sciences Arkhangelsk Russia; ^3^ Northern Arctic Federal University Arkhangelsk Russia; ^4^ Department of Zoology and Fisheries Czech University of Life Sciences Prague Prague Czech Republic; ^5^ Aquatic Systems Biology Unit Technical University of Munich Freising Germany; ^6^ Southern Research Station, Center for Bottomland Hardwoods Research U.S. Forest Service Frankfort Kentucky USA; ^7^ Australian Rivers Institute Griffith University Nathan Queensland Australia; ^8^ Department of Aquatic Zoology Western Australian Museum Welshpool Western Australian Australia; ^9^ CIBIO/InBIO/BIOPOLIS—Research Center in Biodiversity and Genetic Resources University of Porto Vairão Portugal; ^10^ Department of Freshwater Invertebrates Albany Museum Makhanda South Africa; ^11^ Department of Zoology and Entomology Rhodes University Makhanda South Africa; ^12^ CNR Water Research Institute Verbania Italy; ^13^ CBMA—Centre of Molecular and Environmental Biology, Department of Biology University of Minho Braga Portugal; ^14^ Cary Institute of Ecosystem Studies Millbrook New York USA; ^15^ Graham Sustainability Institute University of Michigan Ann Arbor Michigan USA; ^16^ Centro de Investigaciones y Transferencia Santa Cruz (CONICET, UNPA, UTN), Unidad Académica San Julián Universidad Nacional de la Patagonia Austral Santa Cruz Argentina; ^17^ Oklahoma Biological Survey and Department of Biology University of Oklahoma Norman Oklahoma USA; ^18^ Institute of Nature Conservation Polish Academy of Sciences Kraków Poland; ^19^ School of Geography University of Nottingham Nottingham UK

**Keywords:** conservation, diversity, ecosystem services, freshwater mussel, horizon scan, mussel health, threats, unionid

## Abstract

We identified 14 emerging and poorly understood threats and opportunities for addressing the global conservation of freshwater mussels over the next decade. A panel of 17 researchers and stakeholders from six continents submitted a total of 56 topics that were ranked and prioritized using a consensus‐building Delphi technique. Our 14 priority topics fell into five broad themes (autecology, population dynamics, global stressors, global diversity, and ecosystem services) and included understanding diets throughout mussel life history; identifying the drivers of population declines; defining metrics for quantifying mussel health; assessing the role of predators, parasites, and disease; informed guidance on the risks and opportunities for captive breeding and translocations; the loss of mussel–fish co‐evolutionary relationships; assessing the effects of increasing surface water changes; understanding the effects of sand and aggregate mining; understanding the effects of drug pollution and other emerging contaminants such as nanomaterials; appreciating the threats and opportunities arising from river restoration; conserving understudied hotspots by building local capacity through the principles of decolonization; identifying appropriate taxonomic units for conservation; improved quantification of the ecosystem services provided by mussels; and understanding how many mussels are enough to provide these services. Solutions for addressing the topics ranged from ecological studies to technological advances and socio‐political engagement. Prioritization of our topics can help to drive a proactive approach to the conservation of this declining group which provides a multitude of important ecosystem services.

## Graphical abstract

We identified 14 emerging and poorly understood threats and opportunities for addressing the global conservation of freshwater mussels over the next decade. Prioritization of our topics can help to drive a proactive approach to the conservation of this declining group which provides a multitude of important ecosystem services.

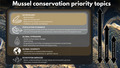

## 1 INTRODUCTION

Freshwater mussels (Bivalvia, Unionida; hereafter, mussels) are important ecosystem engineers in many of the world's rivers, canals, lakes, and ponds, yet globally they represent one of the most imperiled taxonomic groups (Böhm et al., 2021). Mussels can dominate the benthic biomass of rivers (Newton et al., 2011) and their filtration of water, coupled with the creation of biodeposits, plays a key role in transferring suspended material from the water column to the benthos, thus influencing water clarity, primary and secondary production, biogeochemical cycles, and sedimentation rates (Vaughn, 2018). Their shells provide substrate for epiphytes and refuge for macrozoobenthic taxa (Ilarri et al., 2018). The important role of mussels in freshwater ecosystems is also demonstrated by the higher richness of macroinvertebrates in locations with higher mussel densities (Aldridge et al., 2007; Chowdhury et al., 2016; McCasker & Humphries, 2021; Vaughn & Spooner, 2006).

In recent decades, mussels have experienced precipitous declines, with both loss of species (Bogan, 1993) and reductions in abundance (Karatayev et al., 2012). Under current IUCN assessments, 127 of the approximately 300 described species of mussels from North America are considered extinct, possibly extinct, critically endangered, endangered, or vulnerable (IUCN, 2022). Seven of Europe's 16 described mussel species fall within the same risk categories (Lopes‐Lima et al., 2017). While declines have also been reported for the neotropics, afrotropics, Indotropics, and Australasia, lack of comprehensive study means that the conservation status of mussels in these ecoregions remains poorly understood (Lopes‐Lima et al., 2018).

Understanding the drivers of mussel declines has received increasing attention (e.g., Ferreira‐Rodriguez et al., 2019), although research is strongly biased toward Europe and North America. Deforestation and the proliferation of oil palm plantations in Southeast Asia can cause increased channel sedimentation and change environmental characteristics (Gallardo et al., 2018). Habitat alteration, such as through dam construction, can affect sediment transport, temperature, flow, and oxygen regimes, and alter distributions of host fishes (Sousa et al., 2020; Winemiller et al., 2016). Droughts driven through climate change, dam management, and over‐abstraction can lead to mass mortalities (Sousa et al., 2018, 2020; Vaughn et al., 2015). Invasive non‐native species, such as zebra mussels (*Dreissena polymorpha*), can reduce body condition (Sousa et al., 2011) and extirpate local populations of unionids (Lucy et al., 2014). Selective predation by introduced mammals, such as muskrat, has been implicated in population declines and changes in species compositions (Diggins & Stewart, 2000). Over‐harvesting for food and pearls has resulted in population losses in Asia and the Americas (Anthony & Downing, 2001; Zhang et al., 2013), and physical habitat destruction, such as through cattle trampling, is considered a substantial threat in Australia (Walker et al., 2014). The remarkable life history of freshwater mussels exposes them to additional indirect threats: mussels produce parasitic larvae (glochidia) that must successfully parasitize a host (typically a fish) upon which they metamorphose before excysting as juveniles some weeks or months later (Modesto et al., 2018). Initially, the juvenile mussel deposit feeds within the sediment and then transitions toward suspension feeding once its siphons form, resulting in the mussel moving up toward the sediment–water interface (Araujo et al., 2018). This life history means that mussels are vulnerable to declines in their host fishes and deterioration of sediment and water chemistry. While some of the threats to mussels are well known, the cause of many declines remains enigmatic (Haag, 2019).

To halt and reverse declines, there is an increasing interest in projects that focus on propagation and reintroduction, particularly in North America (e.g., Patterson, Mair, et al., 2018) and Europe (e.g., Gum et al., 2011; Patterson, Mair, et al., 2018). The future conservation of mussels on a global scale will depend on a better understanding of the current causes of decline, identifying future threats, and embracing emerging tools. To meet such needs, horizon scanning has developed as a systematic approach to identify and prioritize emerging trends, challenges, and opportunities (Sutherland & Woodroof, 2009) and has successfully been implemented in several fields including conservation (Sutherland et al., 2021) and invasive species (Ricciardi et al., 2017). We assembled a team of 17 freshwater mussel researchers and stakeholders from six continents, representing a wide range of research and management interests. We used consensus building (Box 1) to identify 14 priority topics that could drive a global research agenda for mussel conservation over the next 10 years. The topics are grouped around broad themes and are not in an order of importance.

BOX 1 **CONSENSUS BUILDING AND IDENTIFICATION OF PRIORITY TOPICS**

An international team of 17 researchers and stakeholders active in mussel conservation was assembled from across six continents. Each participant was asked to write at least two short (200–300 words) synopses identifying novel or emerging topics they viewed as either “challenges” or “opportunities” for freshwater mussel conservation in the next 10 years. To ensure maximum inclusion of ideas, participants were encouraged to consult with their network of researchers and practitioners.

In total, 56 synopses were submitted by participants. A Delphi technique was employed to identify the most important topics (Mukherjee et al., 2015), using recommended approaches to anonymity, inclusiveness, and iterative rounds of voting (Sutherland & Burgman, 2015).

Synopses were anonymized and assembled in random order, then circulated to all participants who were asked to rank the synopses in order of importance from 1 (highest) to 56 (lowest). Criteria for ranking were novelty of topic, pervasiveness (scope of influence), and potential impact on mussel conservation in the next 10 years.

The average rank awarded to each synopsis was calculated and circulated to participants prior to meeting.

Participants met online in May 2021 to discuss the topics. Some synopses were agreed to fall outside the scope of the horizon scan and were removed from further consideration. The remaining synopses were discussed. To avoid bias, guidelines for the discussion asked that the original author of a topic synopsis should not be among the first three people to speak about it. Topics that were proposed more than once were combined and four new topics were proposed, resulting in a revised list of 50 synopses.

After these initial discussions, and taking into account any new evidence and information provided at the first meeting, participants were asked to re‐rank the topic synopses, using the same scoring criteria.

The new average rank for each synopsis was again calculated and circulated to participants. The average interquartile range in rankings for a synopsis decreased from 21.2 in round 1 to 15.8 in round 2 (*t*_102_ = 4.66, *p* < .001), reflecting the positive effect of the consensus‐building process.

**FIGURE 1:**
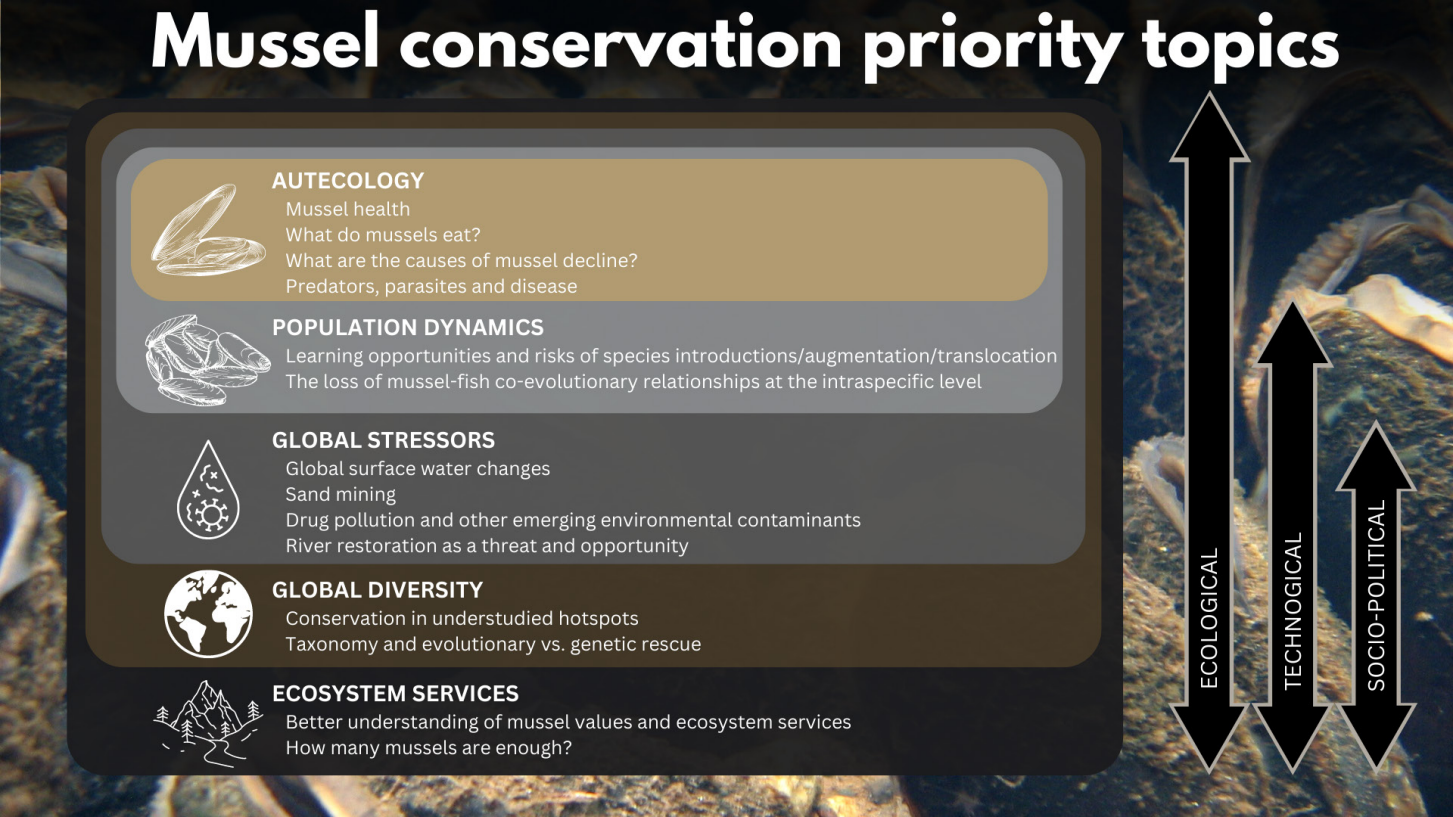
Nesting of the five themes and 14 topics identified through the horizon scanning exercise. Approaches for addressing the themes vary across different scales: Ecological studies are important across all themes; technological approaches are particularly important in addressing topics within population dynamics, global stressors, global diversity, and ecosystem services; socio‐political approaches are needed to address global stressors, global diversity, and ecosystem services. More details of potential solutions are given in each of the 14 topic synopses.

Participants met once more to discuss the new rankings. Again, some similar topics were combined and the highest ranked topics were collectively selected. A total of 14 final topics were agreed upon (Figure 1). These were divided into five broad themes and a working group was formed for each theme to refine and standardize synopses of each final topic.

## 2 THEME: AUTECOLOGY

Four of our topics related to the better understanding of factors affecting mussel autecology, for which future research should particularly focus on ecological studies. In many cases, such studies will need to be directed toward single taxa. The fact that we know so little about the habitat requirements of juvenile mussels and the diet of mussels at any stage in their life history is a fundamental knowledge gap that requires filling.

### 2.1 Mussel health

Discovering causes of mussel declines and developing effective, proactive responses to these factors may often require the ability to assess individual mussel health. Numerous studies have documented mussel responses to specific stressors, such as pesticides, pharmaceuticals, temperature, and pathogens (Bolotov et al., 2018; Castro et al., 2018; Ingersoll et al., 2006; Payton et al., 2016; Waller & Cope, 2019). However, no standard set of biomarkers or other diagnostic tools exist to assess mussel health in response to a wide range of known and unknown stressors. Because mussels are filter feeders, their symbiotic microbiome also may be sensitive to environmental conditions and could provide additional insight about overall health status (Aceves, 2019; McCauley et al., 2021; Mioduchowska et al., 2020; Weingarten et al., 2019). A standardized diagnostic approach specific to mussels is needed that can identify clinical signs of compromised health. For example, molecular tools could be used to nondestructively identify parasites, cellular tools (e.g., hemocyte count, phagocytic activity, lysozyme concentration) could be used to assess immune responses and general stress (Hinzmann et al., 2013), biochemical measures (e.g., glycogen content) can inform on energy stores and identify mussels experiencing stress (Sousa et al., 2011), behavioral measures (e.g., valve closure, gaping, locomotion) can provide an early warning of mussels experiencing unfavorable conditions (Hartmann et al., 2016), and microbiological markers (e.g., microbiome communities) could be used to compare responses of mussels to different diets and growing conditions. Such tools will facilitate the rapid detection of sublethal stress in wild and captive mussels in a wide variety of ecological contexts (Waller & Cope, 2019).

### 2.2 What do mussels eat? Linkages between mussel feeding ecology and conservation

Mussel diets are generally poorly understood. Mussels are considered omnivorous filter feeders that consume bacteria, algae, detritus, zooplankton, dissolved organic matter, and other material (reviewed by Strayer, 2008; Vaughn et al., 2008). However, feeding mechanics and diet may differ substantially among life stages, populations, species, climates, seasons, and habitats. Juveniles appear to be primarily deposit feeders until the filtering apparatus develops (Araujo et al., 2018), but the extent to which adults use deposit versus suspension feeding may vary widely (Raikow & Hamilton, 2001; Weber et al., 2017). Isotopic and fatty acid signatures indicate only modest dietary differences among species in some cases (Fujibayashi et al., 2016; Newton et al., 2013; Weber et al., 2017), but laboratory studies show strong differences among species in food selection and clearance rates (Atkinson et al., 2011; Tran & Ackerman, 2019). Differences in isotopic signatures among rivers indicate that mussels either have nonspecific dietary requirements or they can adapt readily to available food resources in different habitats (Newton et al., 2013). Other approaches, such as DNA metabarcoding, that may alleviate difficulties of stable isotope studies (see Strayer, 2008) are needed to better describe mussel diets. Anthropogenic factors, such as increases in suspended sediment, may negatively affect food acquisition, and such effects appear stronger for juveniles than adults (Tuttle‐Raycraft et al., 2017). Growth was reduced when food resources were dominated by cyanobacteria (Bartsch et al., 2017). These observations suggest that anthropogenic alteration of aquatic food webs and mussel food resources may be important factors in mussel declines. Furthermore, differences in feeding mechanics and dietary requirements among species may explain differential species responses to some types of anthropogenic factors. Isotopic labeling approaches may help to track the uptake of food items at different life stages.

### 2.3 What are the causes of mussel declines?

There is a widespread perception in the conservation community that the causes of mussel declines are reasonably well understood. The effects of dam construction, invasive zebra mussels (*Dreissena* spp.), and a few other factors are well known (Haag, 2012; Strayer & Malcom, 2018). However, support for many other explanations for mussel declines is poor or equivocal (Downing et al., 2010). For example, supporting evidence for the role of sedimentation is limited (Geist & Auerswald, 2007; Haag, 2019; Strayer & Malcom, 2012). Other factors, such as disease, parasites, and the invasive Asian clam (*Corbicula fluminea*), have only recently received serious attention (Brian & Aldridge, 2019; Haag et al., 2021; Richard et al., 2020). Unexplained mussel die‐offs have been documented for over 40 years (Neves, 1987), but it is unknown if these are independent, localized events or parts of a larger pattern of mussel declines. Current conservation approaches are largely motivated by untested ideas about the causes of mussel declines. A better, mechanistic understanding of causes is needed to direct funds in directions that have a better chance of reversing or preventing mussel declines. In particular, rapid response plans should be developed for enigmatic die‐offs so that dying individuals can be collected alongside control animals and preserved appropriately for subsequent autopsy.

### 2.4 Predators, parasites, and disease

Predation has been widely attributed to the localized decline of adult mussels. Studies have focused on both introduced species such a muskrats (*Ondatra zibethicus*; Haag, 2012), feral pigs (*Sus scofra*; van Ee et al., 2020), crayfish (Meira et al., 2019), and coypu (*Myocastor coypus*; Nagayama et al., 2020), and native predators, such as otters (*Lutra lutra*; Zając, 2014). However, the population‐level effects of predators upon mussels have received little quantitative attention. Predators, such as fish (Haag, 2012) and flatworms (Zimmerman et al., 2003), may also be important in preventing juveniles from reaching adulthood in natural populations (Strayer & Malcom, 2018). Endosymbionts can affect mussels throughout their life history, yet parasites and disease are not considered in an IUCN assessment of any unionid mussel (Brian & Aldridge, 2019). Reduced fecundity in mussels has been reported from adults parasitized by trematodes, unionicolid mites (Brian, Dunne, et al., 2021), and ciliates (Lynn et al., 2018), but no parasites of juvenile mussels have been described. Effective conservation of mussels requires a more comprehensive understanding of the roles of predators, parasites, and disease in affecting survival both for cultivation programs within hatchery systems and in natural populations. Without such information, it is impossible identify or prioritize effective management approaches.

## 3 THEME: POPULATION DYNAMICS

The topics identified within the population dynamics theme highlight the important consideration of the interaction that mussels have with their fish hosts (Modesto et al., 2018) and demonstrate the need for integrated approaches to conservation. Solutions to these challenges require fundamental ecological studies which can underpin the development of technologies and best practices, particularly for captive breeding of mussels.

### 3.1 Learning opportunities and risks of species reintroductions, population augmentation, and translocation

Species reintroduction and population augmentation with captively propagated juveniles and translocation of adult mussels are now widely practiced, particularly in Europe and North America, yet often poorly evaluated (Rytwinski et al., 2021). In Europe, the focus of captive breeding has been on the most endangered mussel species such as *Margaritifera margaritifera* and *Unio crassus*, whereas it has been applied to a wider range of species in North America (Gum et al., 2011; Patterson, Jones, & Gatenby, 2018). Most activities typically address a specific conservation goal, such as restoring an extirpated species or removing mussels from construction areas. Unfortunately, published evaluations of these activities are rare, due to their recent advent and the low priority often placed on post‐project monitoring (Strayer et al., 2019). Carefully evaluating the outcomes of these activities can provide critical information about (1) how to conduct them successfully, (2) the causes of mussel declines, and (3) the effects of many environmental factors on mussel populations. Moving mussels among hatchery facilities or natural water bodies also poses risks. Genetic effects of captive breeding efforts have only rarely been monitored (but see Geist et al., 2021). Genetic guidelines for reintroduction and translocation have been proposed (Jones, Hallerman, et al., 2006), but risks of pathogen transmission have only recently been discussed (Brian, Ollard, et al., 2021; Waller & Cope, 2019; Wolf et al., 2019). Pathogens, including trematodes, nematodes, mites, ciliates, bacteria, and viruses can have a wide range of negative effects on mussels (and other species), including catastrophic mortality (Brian & Aldridge, 2019; Richard et al., 2020; Taskinen et al., 1997). In addition, bacterial endosymbionts, that is, *Wolbachia* and *Cardinium*, recently recorded in the threatened *Unio crassus* (Mioduchowska et al., 2020), can significantly increase parthenogenesis and female‐biased sex ratios through feminization, male killing, and cytoplasmic incompatibility. The infection and severity of these endosymbionts are expected to be affected by climate change (Charlesworth et al., 2019). Considering their potential detrimental impacts on host fitness and population demography, the presence of these endosymbionts is of serious conservation concern. Using molecular techniques, populations declining in the wild or maternal stock used in restoration or reintroduction projects must be screened for these endosymbionts. Quarantine and pathogen monitoring are not included in most projects (Brian, Ollard, et al., 2021). We know little about pathogen distribution, modes of transmission, and the susceptibility of recipient populations. Such information is necessary to develop tools and best practice guidelines to effectively mitigate risks of pathogen spread.

### 3.2 The loss of mussel–fish coevolutionary relationships at the intraspecific level

Completion of mussel life cycles depends on the availability of compatible host fish species and lineages. Loss of host fish species, on which larvae (glochidia) attach and metamorphose, may threaten mussel populations. Besides the occurrence of invasive cryptic lineages of freshwater fish (Morais & Reichard, 2018), intentional introductions are particularly common with tens of billions of individuals of varying domestication levels introduced into natural habitats worldwide yearly (Cucherousset & Olden, 2020). These processes inevitably change the ecology, behavior, and immunity of fish individuals, and we lack data on how this affects the ecological and physiological compatibility between mussels and fish. Intraspecific host relationships of mussels show substantial differences in metamorphosis success and developmental times even within individual catchments (Douda et al., 2014; Karlsson et al., 2014; Taeubert et al., 2010). Current conservation practice and host‐fish research must be supplemented by host compatibility testing and conservation strategies going below the species level. Another potential source of emerging mussel–fish relationship threats at the interspecific level can paradoxically be the rapid development of freshwater mussel culture methods (see previous paragraphs). Varying levels of attention are paid to the choice of host fish (or the settings of in vitro conditions) used for glochidia development and associated unwanted selection during culture (Geist et al., 2021). Increasing speed and potentially high‐capacity production of juveniles for extended periods in captivity, or the use of successive exclusively captive‐bred generations could select for traits that are adaptive to captive breeding larval conditions but non‐adaptive to the hosts and their behavior in the wild. These factors highlight the critical need for data to verify whether cultured mussels thrive and reproduce in the long term (Douda et al., 2021). Such verification is an integral part of conservation breeding activities in other taxa (e.g., Davis et al., 2020) but is not yet commonly performed on mussels. Studies identifying patterns in host‐fish suitability are needed to determine adequate management units for mussel–host resources. New technical solutions may enable simpler and more frequent intraspecific host testing in the field and areas with inadequate laboratory infrastructure (Douda et al., 2020). This will also lead to a better appreciation of how short‐term benefits from fish and mussel captive breeding programs might be offset by long‐term impairment of mussel–fish relationships in the wild.

## 4 THEME: GLOBAL STRESSORS

Growing human populations and climate change are prompting water‐engineering responses in anticipation of greater demand and elevated frequency of floods and droughts (Vörösmarty et al., 2010). Mussels are relatively sedentary, and so are vulnerable to changes in hydrology which may come about through both natural process and engineered responses. The specific activity of sand mining can also both destroy and create habitats for mussels, illustrating the combination of threats and opportunities arising from many of the topics identified. The effects of emerging pollutants, such as drugs and micro‐ and nanoplastics, alongside the growing awareness of the positive and negative effects of endosymbionts, deserve greater attention. While we know little about the independent effects of these stressors on mussels, we know even less about how they may interact in combination.

### 4.1 Global surface water changes

Between 1984 and 2015, 90,000 km^2^ of global permanent surface water disappeared while new permanent surface waters covering 184,000 km^2^ were formed (Pekel et al., 2016). Water loss was mainly due to climate change, land use intensification, water abstraction, and channel diversions (Datry et al., 2017). Much of the increase was related to new reservoirs and new lakes in the Tibetan Plateau (Pekel et al., 2016). These changes have large ecological effects, especially for sedentary organisms like freshwater mussels, including the extinction of some species and opportunities for colonization of new habitats. Some freshwater mussel species are highly sensitive to desiccation while others are relatively tolerant, resulting in species shifts in drought‐stricken communities (Vaughn et al., 2015). Similarly, species with traits maximizing dispersal (e.g., using fish hosts that are migratory or widely stocked by humans, or able to thrive in human‐created habitats such as reservoirs and canals) will best take advantage of the new habitats formed. Engineered solutions to cope with water scarcity are particularly alarming, including the implementation of water transfer megaprojects such as China's South‐to‐North Water Transfer Project. These megaprojects are commonly associated with large‐scale agricultural and energy development schemes (Shumilova et al., 2018) in North America, Asia, and Africa, and may be responsible for the introduction of invasive species (Gallardo & Aldridge, 2018) and homogenization of freshwater mussel (and other taxa) communities. Changes in global surface waters present a novel conservation challenge that might need to be actively managed, through translocation of mussels, better regulation of river flow, and the use of remote sensing to assess spatial and temporal changes in hydroperiod in real time (Kissel et al., 2020). In addition, the availability of new anthropogenic habitats (e.g., reservoirs and canals; see Sousa et al., 2021) should also be assessed as refuges or ecological traps for freshwater mussels.

### 4.2 Sand mining

Sand (used here as a generic term that includes any riverine aggregates regardless of particle size) is the second‐most consumed natural resource (after water) on the planet, with 32–50 billion tonnes of sand and gravel extracted annually (UNEP Global Environmental Alert Service, 2014). Demand for sand and other aggregates is expected to increase in coming decades due to rapid human population growth and urbanization, especially in countries with a developing construction industry (Chen, 2017; Gavriletea, 2017). Besides construction, sand and other aggregates are used for land reclamation and have numerous applications in other industries. Sand mining in and near stream channels can cause channel incision, alter riparian zones and change sediment transport (Koehnken et al., 2020), and alter patterns in host fish distribution and abundance (Gavriletea, 2017), resulting in pervasive effects on freshwater biodiversity, especially for benthic species such as mussels (Hartfield, 1993). Positive effects are also possible because aggregate extraction creates novel habitats for mussels—in Europe, gravel pits are commonly colonized by mussels at very high densities (Sousa et al., 2021). There is a growing interest in nature‐friendly design or restoration of gravel pits (Damnjanović et al., 2019), usually aiming at creating habitat favorable for birds, and mussels may benefit from these new anthropogenic habitats (Sousa et al., 2021). Impacts of sand mining on mussels are still poorly known, and should be assessed in the appropriate spatial and temporal dimensions; remote sensing may be useful (Sonter et al., 2018). Impacts of mining should be mitigated as far as possible, perhaps including translocating mussels to unaffected areas.

### 4.3 Drug pollution and other emerging environmental contaminants

The widespread consumption and subsequent release of pharmaceutical and personal care products (PPCPs) to freshwaters have increased at a rate greatly surpassing that of other environmental contaminants; yet PPCPs have seldom appeared in global change discussions (Bernhardt et al., 2017). The biological effects of PPCPs on non‐target organisms, like mussels, can be severe to both the organism's fitness and reproduction, including oocyte maturation, spawning, and parturition (release of glochidia; Gilroy et al., 2020; Richmond et al., 2017). For example, fluoxetine, a commonly prescribed drug to treat disorders such as depression and panic attacks, is reported to artificially induce spawning and disrupt reproduction in mussels (Bringolf et al., 2010). Microplastic and nanoplastic pollution in marine systems has received considerable attention and has demonstrated negative effects on bivalves at the molecular (Canesi et al., 2015), cellular (Paul‐Pont et al., 2016), and organismal level (Li et al., 2020). While it has been demonstrated that freshwater mussels uptake microplastics (e.g., Berglund et al., 2019), little is known about the effects, especially in natural ecosystems. To date, no attention has been given to the effects of nanomaterials on freshwater mussels within natural ecosystems. The consequence of PPCPs, working synergistically, additively, or negatively with other emergent contaminants (e.g., brominated flame retardants, pesticides, and micro/nanoplastics), in mussel conservation is under‐appreciated. Our understanding of the threats to juvenile mussels is limited or nonexistent for most species, and most data come from laboratory exposures to toxicants of captively bred individuals within hatcheries (e.g., Jones et al., 2005) rather than from natural systems. Laboratory studies indicate that early life stages of freshwater mussels may be more responsive to some toxicants than adult mussels (e.g., Cope et al., 2008; Haag et al., 2019).

### 4.4 River restoration as a threat and opportunity

River restoration has become common and is likely to increase in the future (Geist & Hawkins, 2016); for example, the European Biodiversity Strategy for 2030 aims to restore >25,000 km of rivers (European Commission Directorate‐General for Environment, 2021). Restoration covers a wide range of activities, including dam removal, changes to release schedules from reservoirs, adjustments to channel and bed materials, daylighting of buried streams, and management of riparian zones and catchments (Palmer et al., 2014). Goals typically are to restore more natural conditions; stabilize channels; or improve habitat, connectivity, biodiversity, or ecological functioning (Palmer et al., 2014), but the setting of restoration targets is also strongly influenced by human perception and the values for people (Deffner & Haase, 2018). River restoration can benefit mussels by improving connectivity in fragmented populations, restoring host fish populations (Benson et al., 2018; Foley et al., 2017), improving hydrologic and thermal regimes (Palmer & Ruhi, 2019), stabilizing streambeds (Palmer et al., 2014), re‐establishing streambed dynamics (Pander et al., 2019), reducing harmful impacts of non‐native species, and removing contaminants. However, restoration can harm mussel populations by mobilizing sediments formerly held behind dams (Foley et al., 2017; Sethi et al., 2004), increasing scour and other effects of dam removal on downstream habitats (Gangloff, 2013), or increasing dispersal routes for non‐native species (Foley et al., 2017; Paillex et al., 2015). Effects of restoration on mussels have been rarely assessed, and usually over the short term and with low statistical power to detect effects (Barnett & Woolnough, 2021; Heise et al., 2013; Sethi et al., 2004), so effects on mussel populations may not be appreciated, and harmful restoration practices may continue to be applied. To reduce risks and increase benefits, the particular needs of mussels (such as their sedentary nature and the interstitial habits of juveniles) should be considered when planning restoration projects, including more attention to long‐term sediment routing. In addition, mussel populations should be assessed before and after restoration, including long‐term monitoring for at least 10 years, including at least one large, channel‐forming flood and one significant drought.

## 5 THEME: GLOBAL DIVERSITY

The fourth major theme arising from our horizon scan relates to understanding and documenting the global diversity of mussels, for which capacity building is essential, built around the principles of decolonizing conservation. Even in well‐studied regions, recent molecular work is redefining species boundaries (Prié et al., 2012) and providing new perspectives on the most appropriate taxonomic scale for effective conservation (Klunzinger et al., 2021).

### 5.1 Conservation in understudied hotspots

The availability of data, scientific expertise, and conservation resources are distributed unequally across the world. Species richness and endemism are very high in (sub)tropical regions, including the mussel biodiversity hotspots of Southeast Asia and Central America (Graf & Cummings, 2021; Mittermeier et al., 2011), where freshwater biodiversity is declining rapidly (Lopes‐Lima et al., 2018). Given the lack of expertise on these poorly known biodiversity hotspots, collaborations, information‐sharing, and funding are required to develop capacity and establish long‐term conservation projects led by local scientists and which integrate and train early career researchers in freshwater mussel taxonomy, ecology, and conservation. Protecting mussel biodiversity is underpinned by conservation status assessment, requiring species' biological, ecological, and threat data. Despite recent advances in understanding (sub)tropical mussel diversity and ecology, information gaps persist (Bolotov et al., 2020; Pereira et al., 2014; Walker et al., 2014). A scan of the IUCN Red List reveals an almost complete lack of conservation assessments (e.g., South America) or a high number of data‐deficient species (e.g., Africa and Indotropics) in these areas (Lopes‐Lima et al., 2018). Therefore, rapid collection of relevant data is urgent considering the fast rates of decline in mussels (Lopes‐Lima et al., 2021). Novel methods, including environmental DNA (meta)barcoding and underwater drones, have recently emerged, which would improve the speed, reliability, and scope of data collection (Collas et al., 2020; Prié et al., 2021). Additionally, provision of biodiversity data through platforms such as MolluscaBase (http://www.molluscabase.org) or MUSSELp (http://mussel‐project.uwsp.edu) for taxonomy, GBIF (https://www.gbif.org) for distribution, and GenBank (https://www.ncbi.nlm.nih.gov/genbank) or BOLD (https://www.boldsystems.org) for genetic information, making sure that these databases are dynamically cross‐updated, and that identifications are checked and verified, will ensure that data essential for informing conservation reach the right audience (Graf & Cummings, 2021). Integration of biological data across different platforms is challenging and requires collaboration between the database providers. Hypertext links can facilitate navigation between databases.

### 5.2 Taxonomy and evolutionary versus genetic rescue

Species delimitation is important in mussel conservation because most legislation uses species as conservation units. However, several factors can challenge traditional morphology‐based classification (Benson et al., 2022; Klunzinger et al., 2021; Zieritz et al., 2010). Recent molecular re‐evaluations have identified biogeographically restricted taxonomic units of conservation concern, including *Unio mancus* in Europe (Prié et al., 2012) and *Westralunio carteri* in south‐western Australia (Benson et al., 2022; Klunzinger et al., 2021). Conversely, molecular re‐evaluations of three threatened American *Cyclonaias* species demonstrated that they were synonyms of globally secure *C. pustulosa* (Johnson et al., 2018). The situation becomes even more complex when conservation units are deduced within recognized species. Within the Holarctic *Margaritifera margaritifera*, pronounced levels of genetic differentiation were observed at small spatial scales within Europe (Geist & Kuehn, 2005; Geist et al., 2018), whereas the majority of North American populations were all part of one single conservation unit (Zanatta et al., 2018). Without a comprehensive review of the taxonomy of even well‐studied mussel species, using modern molecular tools, we risk overlooking the protection of unrecognized distinct taxonomic units. Within species, there is also the need to prioritize important populations for conservation. Given that species persist through natural selection, an effort has been made to integrate evolutionary concepts into conservation practice (Hendry et al., 2011). However, maintaining neutral genetic variation rather than adaptation has been prioritized in conservation (e.g., Jones, Neves, et al., 2006), ignoring the link between phenotypes and demography; this is concerning when protecting populations. Evolutionary rescue occurs rarely in nature for species with low population size, long generation times, and limited genetic variability, which may be the case in threatened mussels. Conservation plans aim to conserve areas with higher genetic diversity and divergent lineages/populations with unique gene pools. Alternatively, introducing new alleles from elsewhere may improve the fitness of restricted populations by the introduction of new genetic material (i.e., genetic rescue). These measures have been used successfully in small and inbred populations of vertebrates (e.g., Edmands, 2007). Such conservation measures should be approached with caution due to the balance between inbreeding and outbreeding depression by the inclusion of new genes into a population.

## 6 THEME: ECOSYSTEM SERVICES

At the widest scale, our horizon scan identified the growing recognition of mussels as providers of a multitude of ecosystem services as a topic that will be crucial for their long‐term conservation. Only through a robust quantification of the ecosystem services provided by mussels will we be able to set evidence‐based management goals that will maintain fully functioning ecosystems. Moreover, by demonstrating the important roles that mussels play in the world's rivers, streams, ponds, and lakes, we will be better able to engage with the wider public who will become increasingly important contributors to the future conservation of mussels (Clavijo, 2018; Cochero, 2018).

### 6.1 Better understanding of values and ecosystem services provided by mussels

Freshwater mussels provide multiple benefits (ecosystem services) to people, including provisioning services (food for people and other species, medicine, products from shells), regulating services (biofiltration that improves water quality, sequestration of harmful substances, nutrient cycling and storage, habitat and habitat modification for other species, food web support, environmental monitoring), and cultural services (spiritual benefits, ornamentation, conservation values; Zieritz et al., 2022). However, these benefits are likely largely underestimated, undervalued, and unknown to most people, which limits their use as a conservation tool (Strayer, 2017). Efforts are needed to quantify mussel‐provided ecosystem services globally, especially in underexplored areas outside North America, which will also require estimating mussel abundance. In many regions, quantifying direct benefits provided by mussels, such as (functional) food provision throughout Asia (Ke et al., 2011), will be a good starting point. Research is also needed to explore newly recognized benefits such as their potential to serve as early‐warning systems of human diseases and remove pathogens and contaminants from the water (Bianchi et al., 2014). Robust efforts are needed to assign value to mussel services, including both economic and social value, which will require collaboration with social scientists and economists and the use of non‐market and modeling methods (Strayer, 2017). Finally, significant outreach efforts are needed to raise awareness of mussels and their benefits to the general public and decision makers. These efforts could include crowdsourcing projects aided by novel tools and approaches, such as the use of internet data (Jarić et al., 2020; culturomics; Ladle et al., 2016).

### 6.2 How many mussels are enough?

Mussel populations that are abundant and self‐sustaining can best provide ecosystem services to humans. In addition, there are many benefits to both mussels and other species from living in diverse, dense mussel beds including increased condition, higher fertilization rates, higher survival rates, density‐dependent hydrological habitat feedbacks, and enhanced material and nutrient cycling (Atkinson & Vaughn, 2015; Sansom et al., 2020; Terui et al., 2015). How do we know when we have enough mussels? Do we focus on single‐species population viability or do we assess communities? Is there a critical density that ensures successful reproduction, which may be a bottleneck for small, isolated populations? Is there a critical species composition, or age and size distribution? In most regions of the world, historical mussel abundances are largely undocumented but may be approximated through traditional ecological knowledge and other methods. Long‐term monitoring studies that describe how demographics vary over space and time are needed, but traditional measures of mussel populations, such as density and species richness, may not be sensitive enough due to long response times and life spans (Freshwater Mollusk Conservation Society, 2016). Abundance–impact curves, currently used to understand and manage the impacts of invasive species, could be used to estimate critical mussel densities for maintaining mussel function and services (Strayer, 2020).

## 7 CONCLUSIONS

In recent years, declines of freshwater mussels have been documented at the global (Böhm et al., 2021), regional (Zieritz et al., 2018), and local scale (Karatayev et al., 2012). Some of the major drivers of these declines, such as habitat loss (Vaughn & Taylor, 1999), pollution (Strayer & Malcom, 2012), invasive non‐native species (Sousa et al., 2014), and the effects of climate change (Gallardo & Aldridge, 2013) have been reported widely across many freshwater taxa and are well known (Dudgeon et al., 2006). However, our horizon scan identified 14 emerging or poorly understood topics that deserve particular attention for the global conservation of freshwater mussels over the next decade. As such, our scan helps to establish a research agenda for immediate prioritization. The five broad themes into which we categorized our topics can be considered to be nested, with each theme requiring a particular range of ecological, technological, and socio‐political approaches to address them effectively (Figure 1). We did not rank the priority topics in order of importance as different topics are likely to be of interest to different researchers working across different geographies. However, a foundational requirement for successfully addressing all of our topics is to understand the global diversity of freshwater mussels and to establish a robust taxonomy.

Additional important topics were rejected from our horizon scan because it was felt that they lacked novelty or that they would not play an especially important role in mussel conservation over the next decade. Well‐known topics which we felt were important, but which already are subject to considerable research attention, included habitat loss through dam construction and channelization; climate change; fragmentation; declines in host fishes; introduction of invasive non‐native species; predation; and over‐harvesting for food. Topics that we felt may become important over longer timescales, and were worthy of future consideration, included the development of genome editing and gene drives as tools to manage potentially harmful non‐native invasive species; the use of internet data and culturomics to better understand distributions and assess threats; the role and importance of cancers on individuals and populations; and the pros and cons of assisted migration under climate change.

Horizon scanning through the use of consensus‐building techniques has proven to be effective and influential in helping to prioritize conservation efforts and in targeting limited resources toward the most pressing needs (e.g., Sutherland et al., 2022). The Delphi technique requires a formal process of iterative decision‐making between participants that has conventionally used face‐to‐face meetings to facilitate discussion and debate. Due to the Sars‐Cov‐2 pandemic, our entire horizon scan was conducted virtually using an online meeting platform (Zoom) and was found to be an effective process for employing the Delphi framework. While the absence of an in‐person meeting did not allow for the informal discussion of topics during breaks and meals, we advocate the use of virtual horizon scans as an efficient, cost‐effective, and low‐carbon approach that can allow for wider inclusiveness of participation. Horizon scans can help to identify emerging issues that can facilitate pro‐active rather than entirely reactive management responses, thus leading to more desirable outcomes for conservation.

## AUTHOR CONTRIBUTIONS

David C. Aldridge conceived the idea. David C. Aldridge and Isobel S. Ollard organized the workshop and led the writing of the paper. All authors provided initial topic paragraphs, contributed to the workshop, worked in subgroups to produce topic paragraphs for inclusion in the final manuscript, and reviewed and edited the final paper.

## CONFLICT OF INTEREST

The authors have no conflict of interest to declare.

## Data Availability

Data sharing not applicable to this article as no datasets were generated or analysed during the current study.
